# To a Club Patient

**Published:** 1907-10-19

**Authors:** 


### The Lighter Side of Practice.

### TO A CLUB PATIENT.

BY A SADDENED CLUB DOCTOR.

Clubber, as you slowly wander
To the surgery each day,
Tell me, do you ever ponder
On the things you make me say?

Do you sometimes think me lacking
In a sense of all that's right,
When I seem to doubt that "hacking
Cough," and scorn your doleful plight?

When you mourn that "tired feeling,"
Am I slow to sympathise?
Is your sorrowful appealing
Yarn a pack of subtle lies?

Is it fact, or is it fiction?
(You can tell me, if you will),
Is your present sad affliction
Lack of pence; or are you ill?

Stay—you say the Fates assail you
With their customary knocks;
Very well, I will not fail you;
I will put you on the box.

Tell me why you yearn to swallow
Pills of an uncommon size,
With a draught or two to follow,
Which will fill you with surprise.

How is it you think me clever,
When I turn a hurried eye
On your outstretched tongue, and never
Spot your ills, or even try?

Is it that my drugs are noted
For their all-pervading scent,
While the bottle's base is coated
With a muddy sediment?

Have you never once suspected,
When you sought my cheap advice,
That you are a bit neglected;
That I'm dear at any price?

Clubber, sometimes I have scorned you,
Heard your tales with scant belief;
Anyway, I now have warned you;
And I feel some slight relief. F. G. L.

